# Macular edema is a rare finding in untreated vitreoretinal lymphoma: small case series and review of the literature

**DOI:** 10.1186/s40942-017-0067-x

**Published:** 2017-04-24

**Authors:** Elisa Carreras, Diva R. Salomão, Jeroni Nadal, Sejal R. Amin, Harish Raja, Thomas J. Grube, Ryan L. Geraets, Patrick B. Johnston, Brian P. O’Neill, Jose S. Pulido

**Affiliations:** 1 Department of Ophthalmology, Mayo Clinic, 200 First Street, SW, Rochester, MN 55905 USA; 2 Barraquer Institute, Barcelona, Spain; 3 Universitat Autònoma de Barcelona, Barcelona, Spain; 4 Departments of Ophthalmology and Anatomic Pathology, Mayo Clinic, Rochester, MN USA; 5 Grube Retina Clinic, Mandan, ND USA; 6 Ophthalmology LTD, Sioux Falls, SD USA; 7 Division of Hematology, Mayo Clinic, Rochester, MN USA; 8 Department of Neurology, Mayo Clinic, Rochester, MN USA

## Abstract

**Background:**

To determine the occurrence of macular edema (ME) in vitreoretinal lymphoma (VRL).

**Methods:**

Retrospective analysis of 17 patients (31 eyes) with VRL. A review of the literature was done as well.

**Results:**

Nine patients (15 eyes) had fluorescein angiography and/or optical coherence tomography at presentation. In the ME group (six eyes of four patients), three patients (five eyes) had prior chemotherapy and radiation. Excluding eyes with radiation retinopathy (three eyes), rate of ME was 25% (3/12). When two unirradiated fellow eyes of eyes with radiation retinopathy were also excluded, ME rate was 10% (1/10). Excluding the eyes with intraocular surgery, the rate of ME was 0%. In the group without ME (nine eyes of six patients), one patient (one eye) was treated with chemotherapy and radiation and three patients (five eyes) with chemotherapy. Review of the literature showed that the ME was found between 2 and 60% of cases, but most of the cases with ME had prior interventions.

**Conclusions:**

Macular edema in VRL is not uncommon but usually related to prior interventions. Macular edema as an initial presentation of VRL is rare.

## Introduction

Vitreoretinal lymphoma (VRL) is a rare form of non-Hodgkin central nervous system lymphoma (CNS-L). Malignant diffuse large B cell is the most common form, although rarely, a T-cell form occurs as well [1, 2]. These cells invade the vitreous and retina, including the subretinal and sub-RPE (retinal pigment epithelium) space.

Ocular involvement can precede (primary VRL), occur in tandem with, or follow CNS disease (secondary VRL) [3]. Therefore, patients with CNS-L will go on to develop ocular involvement 15–25% of the time; while in those patients with primary VRL, upwards of 65–90% of patients will develop cerebral disease [4, 5]. Occasionally, VRL is associated with systemic non-Hodgkin lymphoma [6, 7].

VRL usually affects elderly patients between the sixth and seventh decades of life but can occasionally occur in younger patients, though these patients tend to be immunocompromised. It is bilateral in 60–90% of cases but is sometimes asymmetric at the time of presentation [6]. Floaters and blurred vision are the most typical symptoms. Clinical findings can vary widely, and the condition may masquerade as a bilateral posterior uveitis.

The most common presentation is vitreous invasion by clumps of cells. Solitary or multifocal sub-RPE lesions can occur with or without vitreous involvement. Less frequent manifestations include: macular edema (ME), iridocyclitis, optic neuropathy, vasculitis, retinal detachment, and retinal hemorrhage [5, 8].

Macular edema is a nonspecific finding of uveitis secondary to blood-retinal barrier disruption as a result of inflammatory mediators. It is frequently present in vitreous inflammatory diseases such as intermediate uveitis [9], but it is also seen in anterior, posterior, and panuveitis; it is the main cause of visual impairment in many cases of uveitis [10, 11]. Interestingly, ME does not appear to be a characteristic finding in VRL, though there are few studies and some use only funduscopic determination [12–15]. Fluorescein angiography and ocular coherence tomography (OCT) are very good at detecting ME in VRL. The purpose of the present review is to determine the occurrence and behavior of ME at the time of the initial presentation of VRL.

This is a retrospective study of all patients diagnosed with VRL within a 5-year period (January 2005–January 2010) at Mayo Clinic, Rochester, Minnesota, USA. All patients who were included in this study had tissue diagnosis at Mayo Clinic within an average of three weeks of initial presentation.

After Mayo Clinic IRB approval, we reviewed all cases diagnosed with VRL that had tissue confirmation. We then selected those that had fluorescein angiography (FA) and/or OCT at their initial visit at Mayo. These were classified in two groups depending on the presence or absence of ME. In both groups, we analyzed the best corrected visual acuity (BCVA) as well as the relevant past medical, pharmacological, radiotherapy, and ocular history, including prior intraocular procedures. Intraocular findings, including the severity of vitreous cells and retinal involvement (intraretinal or subretinal) at the time of initial examination, were also recorded. Those eyes with FA and/or OCT had their charts re-evaluated at 6 and 12 months’ time.

The OCT was analyzed with careful attention to the appearance of intraretinal cysts or fluid, retinal thickness, flattening of the foveal depression, decrease of intraretinal reflectivity, and serous retinal detachment as a result of fluid accumulation in the OCT in six radial line scan images.

Any detectable ME on OCT was classified in three groups (i.e., cystoid, diffuse, or serous detachment) as described in the literature [16–18]. Briefly, cystoid ME is characterized by the presence of low-reflective intraretinal spaces separated by thin retinal tissue. Diffuse ME has an increased macular thickness with a spongy appearance of the retinal layer. Finally, serous detachment is defined as the presence of serous retinal detachment, epiretinal membrane, or vitreoretinal traction.

To classify the angiographic ME, we used the grading system of Yannuzzi et al. [19, 20]. ME was classified in two patterns: cystoid or diffuse. The cystoid macular pattern demonstrated a demarcated petaloid pattern of hyperfluorescence, whereas the diffuse pattern showed a late spread of leakage in a poorly demarcated area in the foveal or perifoveal region. Grade 0 corresponded to the absence of perifoveal hyperfluorescence; grade 1 referred to incomplete perifoveal hyperfluorescence; grade 2 referred to mild 360° hyperfluorescence; grade 3 was characterized by moderate 360° hyperfluorescence with the area of hyperfluorescence being 1 disc diameter across; and grade 4 referred to severe 360° hyperfluorescence with the hyperfluorescent area having a minimal cross-sectional diameter of 1.5 disc diameter.

Subsequently, a review of the English literature was performed as well. Keywords used included vitreoretinal lymphoma and intraocular lymphoma.

## Cases at Mayo Clinic

During the 5-year study period, 17 patients (31 eyes) were histopathologically diagnosed with VRL, but only 15 patients required imaging at the first visit. In eight patients (47%), VRL preceded the CNS-L; whereas in nine patients (53%), VRL occurred in patients with the diagnosis of CNS-L. Sixteen (52%) were right eyes and 15 (48%) were left eyes.

**Fig. 1:**
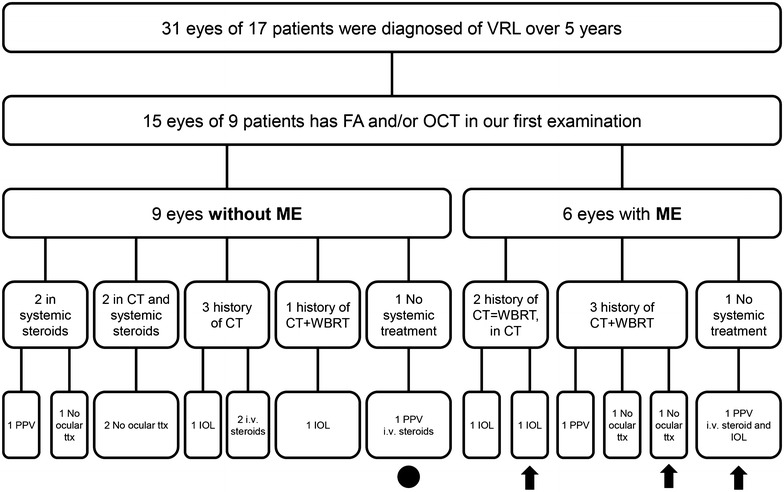
Flowchart of patients with vitreoretinal lymphoma with and without macular edema. Flowchart depicting the selection of vitreoretinal lymphoma (VRL) patients at Mayo Clinic. Fluorescein angiography (FA), optical coherence tomography (OCT), macular edema (ME), treatment (ttx), intravitreal (i.v.), cataract surgery with intraocular lens implantation (IOL), pars plana vitrectomy (PPV), whole-brain radiotherapy (WBRT), chemotherapy (CT). The point marks the eye that developed ME during our follow-up. *Arrows mark* eyes without radiation retinopathy (RR)

The median age for all 17 patients diagnosed with VRL was 66 years (range 54–80 years) at presentation. Nine patients (53%) were female and eight (47%) were male (Table 1). Fourteen patients (82%) had bilateral presentation and three (18%) had unilateral presentation. Nine patients (15 eyes) were imaged with FA and/or OCT at the first examination (Fig. 1).

**Table 1:** Systemic history of patients studied

Patient	Sex	Systemic disease	VRL type	CNS type	Age at CNS-L dx	CT as only ttx	CT previous WBRT	Latency from CT to WBRT	WBRT	cGY	Latency from WBRT to VRL	Post CT	Last dose of CT	CT in VRL dx	CNS involves. at VRL dx
1	M	No	S	CNS-L	50	–	HD-MTX	2 months	Brain ON	3600 Brain 2520 ON	8 months	No	10 months	No	Yes
2	F	No	P	CNS-L	72	–	–	–	–	–	–	–	–	No	No
3	F	HBP	S	CNS-L	65	–	CHOP	2 months	Yes	?	36 months	No	29 months	No	No
4	M	Spindle cell sarcoma	S	DLCB	58	–	CHOP	?	Yes	?	132 months	MTX RT ICE	–	Yes	No
5	M	Steven Johnson	S	CNS-L	63	–	CHOP, RT	10 months	Yes	?	16 months	No	27 months	No	Yes
6	F	HBP; Breast cancer	S	CNS-L	67	HD-MTX	–	–	–	–	–	–	3 months	No	Yes
7	F	HBP	P	CNS-L	61	HD-MTX Steroids	–	–	–	–	–	–	–	Yes	Yes
8	F	HBP;DSL;HA; prostate cancer	P	CNS-L	73	Steroids	–	–	–	–	–	–	–	Yes	Yes
9	M	HA	S	CNS-L	79	CHOP MTX	–	–	–	–	–	–	36 months	No	Yes

The dose and tissue irradiated was only known in one patient because the other patients were treated outside Mayo Clinic, and no reports were available

*HBP* high blood pressure, *DSL* dyslipidemia, *HA* heart attack, *P* primary, *S* secondary, *CNS-L* central nervous system lymphoma, *DX* diagnosis, *TTx* treatment, *CT* chemotherapy, *HD-MTX* high-dose methotrexate, *MTX* methotrexate, *RT* rituximab, *T* temozolomide, *CHOP* cyclophosphamide plus hydroxydaunorubicin plus vincristine plus prednisone, *ICE* ifosfamide plus carboplatin plus etoposide, *WBRT* whole-brain radiotherapy

### ME group

Six eyes (four patients) had ME by FA and/or OCT at the first examination, representing 40% of the imaged VRL patients (6 of 15) (Table 2).

**Table 2:** Ocular history and examination of eyes studied

Patient	Eye	Age at VRL DX	Intraocular procedure	Latency from I.P. to VRL DX	Symptoms	BCVA	AC	Lens	Vitreous	VRL with retinal involvement	Other retinal signs	OCT	FA
1	OS	57	PPV	1 month	BV	20/40	1 + cells 1 + haze	1 + NS	3 + cells	Yes (subretinal)	Hard exudates	Diffuse ME	Diffuse ME
2	OD*	72	PPV I.v. steroids	2 months	No	20/25	No cells	2–3 + NS	No cells	No	–	No ME	–
	OS	72	PPV I.v. steroid IOL	11 and 5 months	BV	20/25	No cells	pcIOL	Rare cells	No	–	Cystoid ME	–
3	OD	68	No	–	BV	20/30	Trace cells	1 + NS 1 + C	Trace cells	Yes (subretinal)	CWS, intraretinal hemorrhage	No ME	Diffuse ME
	OS	68	No	–	BV, F	20/50	Trace cells	1 + NS 1 + C	1 + cells	Yes (retinal)	–	Diffuse ME	Diffuse ME
4	OD	69	IOL	1 month	BV	20/150	No cells	pcIOL with trace PCO	3 + cells 3 + haze	No	–	–	Diffuse ME
	OS	69	IOL	2 months	BV	20/60	No cells	pcIOL with trace PCO	3 + cells 3 + haze	No	CWS	–	Diffuse ME
5	OD	66	IOL	3 months	BV, F	20/200	No cells	pcIOL	3 + cells	Yes (subretinal)	–	No ME	–
6	OD	69	I.v. steroids	1 month	BV, F	20/70	No cells	Trace NS	1 + cells	No	–	No ME	No ME
	OS	69	I.V. steroids	1 month	BV, F	20/60	No cells	Trace NS, Trace PSC	Trace cells	Yes (subretinal)	–	No ME	No ME
7	OD	61	No	–	BV	20/20	No cells	1 + NS 1 + C	2 + cells	No	ERM	No ME; ERM	No ME
	OS	61	No	–	BV	20/25	No cells	1 + NS 1 + C	1 + cells	No	ERM	No ME; ERM	No ME
8	OD	73	No	–	BV, F	20/25	2 + Flare	2 + NS	3 + cells	No	–	No ME; ERM	No ME
	OS	73	PPV	5 months	BV, F	HM	4 + Flare	2–3 + NS	2 + cells	Yes (subretinal)	–	No ME; ERM	No ME
9	OD	80	IOL	168 months	BV, F	20/25	Trace cells, trace flare	pcIOL	1 + cells	No	–	No ME	No ME

*OD* right eye, *OS* left eye, *DX* diagnosis, *IP* intraocular procedure, *IV* intravitreous, *IOL* intraocular lens implantation, *PPV* pars plana vitrectomy, *BV* blurred vision, *F* floaters, *BCVA* best corrected visual acuity, *AC* anterior chamber, *NS* nuclear sclerosis, *C* cortical, *PSC* posterior capsule sclerosis, *pcIOL* posterior chamber intraocular lens, *PCO* posterior capsule opacification, *OCT* optical coherence tomography, *ME* macular edema, *ERM* epiretinal membrane, *FA* fluorescein angiography

The median age in this group was 69 years (range 57–72 years). Two patients were female (50%) and two were male (50%). Two were right eyes (33.3%) and four were left eyes (66.7%).

Only one patient had high blood pressure and was on medical therapy and none had diabetes mellitus. Four eyes had previous intraocular surgery (two had cataract surgery, and two had pars plana vitrectomy).

Three patients (75%) had secondary VRL at the time of diagnosis of CNS-L; two had prior CNS-L and one had diffuse large B-cell lymphoma with CNS involvement diagnosed 3–7 and 11 years prior to their evaluation, respectively. One patient (25%) developed CNS-L 5 months after our examination (primary VRL).

All three patients with previous CNS-L had been previously treated with whole-brain radiotherapy (WBRT). The radiation details of only one patient (patient 1) were known to us because the other patients were treated elsewhere. Additionally, all three patients had been treated with chemotherapy (CT) before the WBRT. One patient (patient 4) had post-radiation CT and was still on a maintenance dosage of intravenous methotrexate at the time of evaluation.

Patient 2 had primary VRL and had no prior treatment with CT or WBRT. However, he had undergone pars plana vitrectomy (PPV) with intravitreal corticosteroid injection and cataract surgery in the left eye, 11 and 5 months before our examination, respectively.

The clinical presentation was blurred vision (5 of 6) and blurred vision plus floaters (1 of 6) for a median of 3 months (range 1–9). The median BCVA was 20/45 (range 20/25–20/150). Ophthalmologic signs included: a median anterior chamber cell grade of 1+ (range 0–3+), a median vitreous cell grade of 3+ (range rare-3+), subretinal infiltrates (2 of 6), intraretinal infiltrates (1 of 6), cotton wool spots (2 of 6), intraretinal hemorrhages (1 of 6), and perimacular hard exudates (1 of 6) (Table 2).

**Fig. 2:**
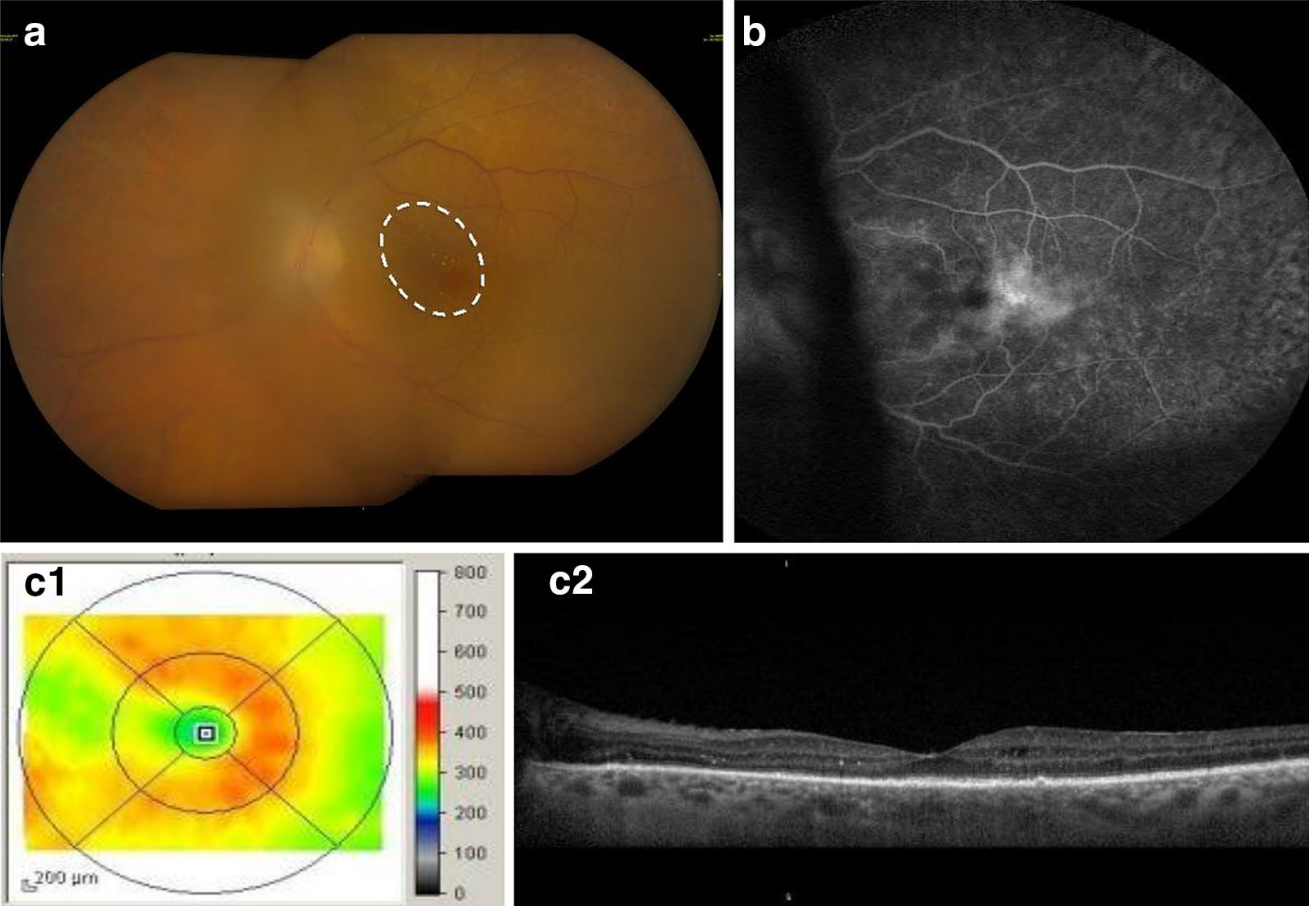
Fundus photograph, FA, and OCT showing diffuse macular edema in a patient with radiation retinopathy and a history of WBRT, CT, and PPV (patient 1). Diffuse macular edema in right eye due to radiation retinopathy in patient with primary VRL with a history of WBRT and chemotherapy and pars plana vitrectomy (patient 1). The fundus examination showed 3+ cells in the vitreous, macular edema and hard exudates nasal to the fovea (line with dashes), and peripheral subretinal mottling and vessels sheathing 360° (**a**). The FA presented extrafoveal diffuse macular edema grade 1 (**b**) and diffuse extrafoveal edema by OCT (**C1**, **2**)

**Fig. 3:**
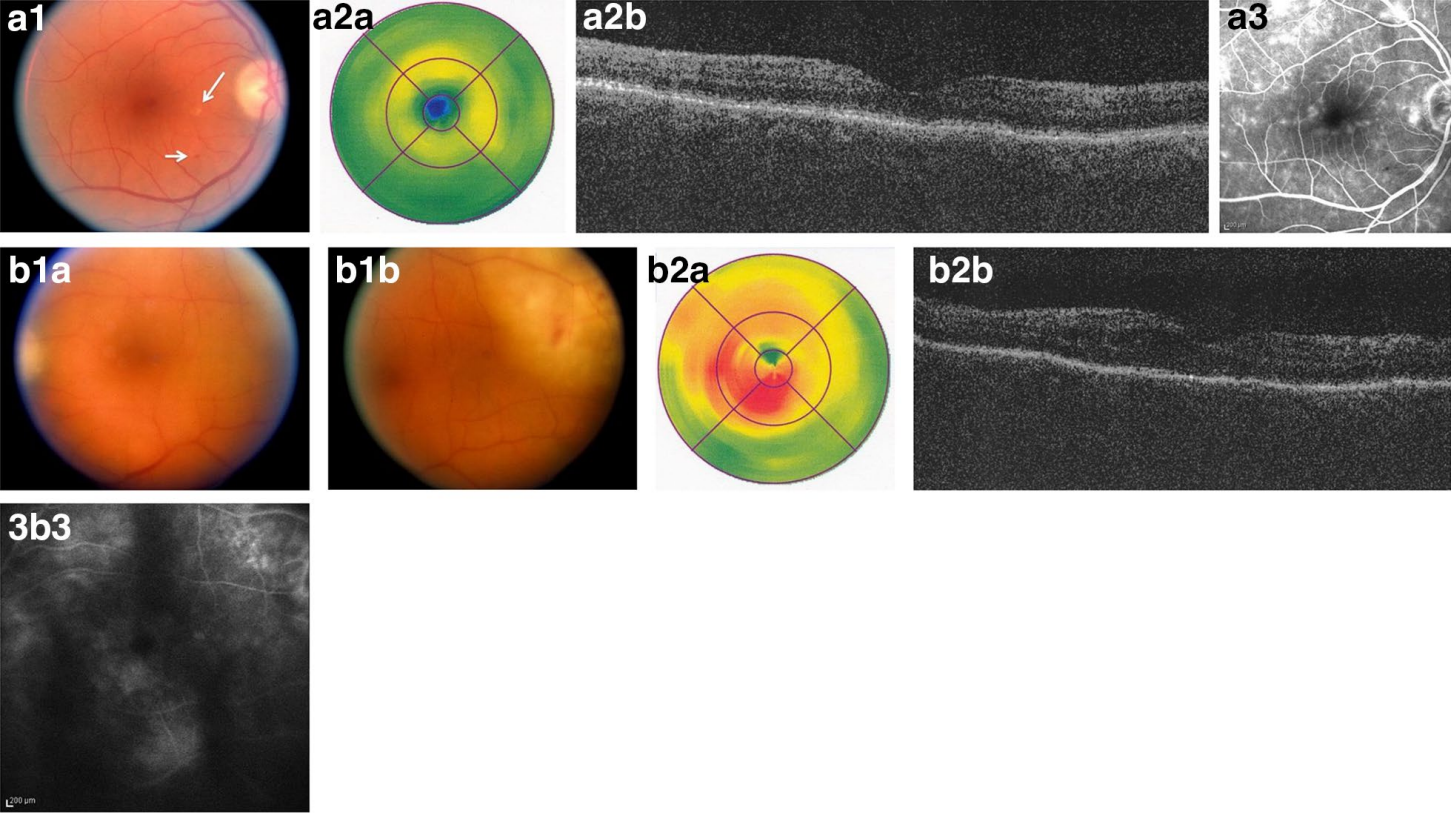
Fundus photographs, FA, and OCT of eye with radiation retinopathy and fellow eye in a patient with history of WBRT and chemotherapy (patient 3). Diffuse macular edema in both eyes, right eye due to radiation retinopathy, in patient with a secondary VRL with a history of WBRT and chemotherapy and no intraocular procedures (patient 3). The fundus examination showed cotton wool spots (*long arrow*) and intraretinal hemorrhage (*short arrow*) in the right eye (**a1**) as well as an intraretinal infiltrate superotemporal to the macula (**b1a, b**), which could be the cause of the ME of this eye (eye without signs of RR). The OCT was negative for ME in the right eye (**a2**) and showed an extrafoveal diffuse ME in the left eye (**b2**). However, the FA presented a diffuse ME in both eyes, grade 1 in right eye and grade 3 in left eye (**a3, b3**)

However, mixed lesions in the fundus (i.e. cotton wool spots, intraretinal hemorrhages, and hard exudates) were found in three patients (3 of 6 eyes) with ME who had received WBRT at 8, 36, and 132 months before presentation (Figs. 2, 3). For this reason, we could not rule out radiation retinopathy (RR) as an etiology of ME in these three eyes. Thus, excluding eyes with RR, the rate of ME was 25% (3 of 12). However, two additional eyes were the fellow eyes of those with definite RR (in which no signs of RR were found). Excluding these as well, the rate of ME in eyes with VRL was 10% (1 of 10) (Fig. 3). Looking at it differently, of five patients without WBRT, 1 of 9 (11.1%) eyes had ME.

Of these three eyes with ME but without signs of RR (or which were not fellow eyes of those with RR), one eye did not have WBRT but did have a history of PPV and cataract surgery history, one had cataract surgery only, and one did not have any intraocular procedures before our examination. The clinical presentation in these eyes was blurred vision (2 of 3) or blurred vision plus floaters (1 of 3) for a median of 2 months (range 2–6). The median BCVA was 20/50 (20/25–20/150), and ophthalmologic signs included a median anterior chamber cell grade of 0 (range 0–trace), a median vitreous cell grade of 1+ (range rare-3+), and intraretinal infiltrates (1 of 3).

Two eyes without RR (or fellow eyes of those with RR) were imaged with FA, showing extrafoveal, grade 1 macular leakage in one of the eyes (1 of 2) and grade 3 macular leakage in the other eye (1 of 2). Two eyes also had OCT done with extrafoveal cystoid ME in one eye (1 of 2) and extrafoveal diffuse ME in the other eye (1 of 2). In the one eye which was imaged both with FA and OCT, there was no discrepancy between the tests to detect diffuse ME. All three eyes with RR underwent imaging with FA and were found to have focal diffuse grade 1 macular leakage. Two eyes with RR were imaged with OCT, and one eye showed extrafoveal diffuse ME (1 of 2), while the other eye (1 of 2) was negative for ME changes. Notably, the FA of this eye was positive for extrafoveal diffuse grade 1 ME. Therefore, there was a discrepancy between FA and OCT to detect ME in half eyes with RR.

Two patients (three eyes) had OCT after 1-year follow-up. In one patient, after PPV in the left eye and intravitreal methotrexate and rituximab injections in both eyes and CT, the ME persisted in the right eye (eye without RR) and was chronic in the left eye (eye with RR) (patient 4). In the left eye of the other patient, without history of WBRT, after CT treatment as a result of CNS-L presentation 5 months after our first examination and no intraocular procedures, ME was worse (patient 2).

### Review of the literature

Macular edema is the result of breakdown of the inner endothelial blood-retinal barrier, developing an increase of retinal vascular permeability, which promotes the accumulation of fluid inside the retinal tissue [21]. This vasogenic effect can be modified in a wide variety of pathologic or pharmacologic conditions, systemic or intraocular [21, 22].

Reviewing the English literature, there are a few authors that describe the incidence of ME in VRL [12–15] (Table 3). The rate varies widely from 2.46% to 66.6% of cases. Patients with VRL who present ME also can have other possible sources of ME, such as antecedents of whole-brain radiotherapy (WBRT), chemotherapy (CT), epiretinal membranes, and/or intraocular procedures [12–15, 23, 24].

**Table 3:** Summary of bibliography

Author	No. patients with VRL	No. eyes with VRL	No. eyes with macular oedema (%)	Total no. of eyes with prior treatment
Cassoux et al. [12]	44	81	2 (2.46%)	3 had PPV
Velez et al. [13]	17	31	6 (19%)	4 had cataract surgery 1 had PPV
Fardeau et al. [14]	53	?	6 patients (11.3%)	?
Turaka et al. [15]	8	10 with FFA 3 with OCT	6 (60%) 2 (66.6%)	50% CT 25% RT 25% CT + RT
Jang et al. [23]	5	5	2 (40%)	1 eye was a secondary VRL, so we assume that had previous CT and/or RT
Saito et al. [24]	20	26	3 (11.53%)	2 eyes had ERM
Our series	9	15	6 (40%)	5 eyes had CT + RT 4 eyes had intraocular surgery

*VRL* vitreoretinal lymphoma, *No* number, *PPV* pars plana vitrectomy, *CT* chemotherapy, *RT* radiotherapy, *ERM* epiretinal membrane

Turaka et al. [15] describes the highest percentage of ME being 60% (6 of 10 eyes) by FA and 66.6% (2 of 3 eyes) by OCT, whereas systemic lymphoma was treated with CT in 50% of patients, external beam radiotherapy in 25%, and combined CT and radiation in 25%. However, there are no specifics described stating that patients with ME had any history of these conditions. In other studies, prior radiation or CT history is not described.

Radiation retinopathy (RR) has been described after radiation treatments of ocular, orbital, and intracranial tumors several months or years after [25–27]. Retinal vascular endothelial cells are damaged by radiation and develop retinopathy [28]. Macular edema is the earliest clinical presentation, followed by hard exudates, microaneurysm, telangiectasia, hemorrhages, neovascularization, and tractional retinal detachment [29]. A dose >50 Gy is associated with RR development. However, there are RR cases reported with doses of <35 Gy [30–32]. Moreover, it has been reported that CT accompanying the radiation treatment can accelerate RR due to retinal vascular damage as happens in diabetic and hypertensive patients [31–33].

Saito et al. [24] describes 11.53% of ME in PVRL (3 of 26 eyes), but history of previous intraocular procedures is not specified. However, 2 of 3 eyes (1 patient) presented epiretinal membranes in the initial presentation; therefore, that could be considered a risk factor to develop macular oedema.

Velez et al. [13] shows 19% (6 of 31 eyes) with cystoid ME. However, four eyes in this study had previous cataract surgery and one had pars plana vitrectomy (PPV), suggesting that a disruption of the anterior-posterior chamber interface predisposes to the development of inflammatory signs such as ME. Cassoux et al. [12] described the lowest percentage of ME of all studies at a rate of 2.46% (2 of 81 eyes) by FA. In this population, three eyes had prior PPV, but exactly which eyes had ME was not clear. Thus, we cannot tell if there is any association with prior surgery in this series.

Considering all of the risk factors for ME, the rate of ME in the setting of VRL is not uncommon. However, most of the cases are related with risk factors WBRT, CT, epiretinal membranes and/or intraocular surgery. Thus, it is difficult to determine the exact incidence of ME due to VRL, per se, but reflects that it possibly is an uncommon sign in eyes without a history of systemic or intraocular interventions.

## Conclusions

In conclusion, although ME is observed predominantly in disorders of the vitreous body, it appears that ME is not a main characteristic of VRL. Furthermore, in cases with marked vitreous inflammation with sheets and clusters of cells without ME as well as good visual acuity, one of our principal differential diagnoses should be VRL. However, a meticulous systemic and intraocular history must be evaluated in all patients since, in patients with prior intervention, ME may be present.

## Abbreviations

ME: macular edema
VRL: vitreoretinal lymphoma
CNS-L: central nervous system lymphoma
OCT: ocular coherence tomography
FA: fluorescein angiography
WBRT: whole-brain radiotherapy
CT: chemotherapy
PPV: pars plana vitrectomy
RR: radiation retinopathy
